# Consultation time and communication patterns in outpatient care: an observational study in South Korea

**DOI:** 10.1186/s12913-025-13431-z

**Published:** 2025-08-30

**Authors:** Minjung Lee, Bumjo Oh, Myoungsoon You

**Affiliations:** 1 School of Nursing, Yale University, 400 West Campus Dr, Orange, Connecticut, 06477 USA; 2 Department of Family Medicine, Seoul National University College of Medicine, 103 Daehak-ro, Jongno-gu, Seoul, Republic of Korea; 3 Department of Family Medicine, Seoul Metropolitan Government-Seoul National University Boramae Medical Center, 20, Boramae-ro 5-gil, Dongjak-gu, Seoul, Republic of Korea; 4 Department of Public Health Science, Graduate School of Public Health, Seoul National University, Gwanak-ro, Gwanak-gu, Seoul, Republic of Korea

**Keywords:** Patient-physician communication, Roter Interaction Analysis System (RIAS), Consultation time, Patient-centered care, South korea, Outpatient

## Abstract

**Background:**

Patient-centered care (PCC) has become a global standard for improving communication and health outcomes. However, in time-pressured clinical settings, especially in high-volume outpatient systems such as South Korea’s, the implementation of PCC remains challenging. While consultation time is often cited as a key barrier, few studies have examined how actual communication patterns relate to consultation duration using observational methods.

**Methods:**

We analyzed 510 audio-recorded outpatient consultations from a secondary-level public hospital in Seoul, South Korea, collected between 2016 and 2018. Communication was manually coded using the Roter Interaction Analysis System (RIAS), a validated method for categorizing each utterance - defined as a single, complete thought or clause - into instrumental (task-oriented) or affective (socio-emotional) domains. Cluster analysis was then used to identify consultation-level communication patterns based on the distribution of RIAS codes. Multinomial logistic regression was conducted to examine associations between communication patterns and consultation time, visit type, physician experience, and other contextual factors.

**Results:**

Three distinct communication patterns were identified: biomedical (physician-led and task-oriented), biopsychosocial (a balanced mix of instrumental and affective communication), and consumerist (patient-driven, information-seeking with limited emotional exchange). The biopsychosocial pattern was the most common and occurred in the shortest consultations (mean = 4.07 min). The biomedical pattern occurred in the longest consultations (mean = 6.78 min) and was characterized by a predominance of physician talk, frequent biomedical questions and directives, and a higher rate of interruptions initiated by the physician. The consumerist pattern appeared more frequently among physicians with longer clinical careers. Multinomial logistic regression showed that follow-up visits (OR = 0.49, 95% CI: 0.24 to 0.56) and shorter consultation time (OR = 0.89, 95% CI: 0.80 to 0.95) were associated with the biopsychosocial pattern. Physicians with longer medical careers were more likely to exhibit the consumerist pattern (OR = 1.08, 95% CI: 1.01 to 1.18).

**Conclusions:**

Communication patterns are not solely determined by consultation length. Patient-centered interactions can take place within brief consultations, highlighting the need to tailor communication training and care delivery strategies to realistic time constraints in high-volume clinical settings.

## Introduction

Patient-centered care (PCC) represents a shift from the traditional biomedical, disease-oriented, and asymmetrical provider-centered paradigm to a model that prioritizes interventions and treatments that are respectful of and responsive to patients’ individual characteristics, needs, preferences, and values [1–3]. At its core, PCC transforms the patient’s role from a passive recipient to an active participant in healthcare decisions, aligning individual preferences with clinical expertise [3]. Widely recognized as a hallmark of high-quality care, PCC has been associated with improved health outcomes, including greater patient satisfaction, enhanced quality of life, and better self-management, making it a cornerstone of modern medical practice [4–6]. Originating in Western countries in the 1960s, PCC has gained traction globally, with significant advancements in regions such as the UK, Australia, Europe, and the USA, where it is regarded as a healthcare priority and embedded in policy initiatives [7, 8]. However, research on the implementation and effectiveness of PCC in Eastern or Asian countries, including South Korea, remains relatively limited [9, 10].

In South Korea, the patient population includes a growing number of individuals with chronic and complex conditions, yet this complexity is often managed through high-frequency, short-duration visits rather than extended consultations [11–13]. One of the challenges of patient-centered care is consultation time. There is ongoing debate about whether longer consultations directly improve outcomes or whether the physician’s communication style and approach play a more decisive role [14–16]. Shorter consultations have been linked to limitations in problem identification, fewer preventive measures, reduced discussions on lifestyle or psychosocial issues, and an increased likelihood of antibiotic prescriptions [17]. In South Korea, the commonly used phrase “three hours waiting, three minutes consultation” reflects public concerns regarding the brevity of medical visits and their impact on patient experience [18]. The average consultation time in South Korea general hospitals ranges from 6.2 to 7.4 min, which is significantly shorter than the averages in the United States at 21 min and the United Kingdom at 9 min. South Korea’s consultation times are more comparable to those in lower resource settings such as Indonesia and Tanzania [19].

These time constraints pose challenges for patient-centered communication and raise concerns about the quality of care and the depth of patient engagement in clinical decision making. Despite the growing burden of chronic diseases in South Korea, outpatient care remains largely fragmented, with patients frequently visiting multiple providers for single-issue consultations, often within a high-volume, short-visit system [20]. The availability of private-sector providers and patient preferences for large hospitals contributes to frequent outpatient utilization [21]. This structural context, where even patients with chronic conditions may receive brief and episodic care, presents unique challenges and opportunities for improving patient-physician communication. This study was conducted at a secondary-level general hospital in South Korea, where outpatient consultations in the internal medicine and surgery departments are typically brief and highly structured [22].

In South Korea, there is a growing movement to establish a patient-centered healthcare system and integrate PCC into practice [23]. The Ministry of Health and Welfare has set a policy goal to provide patient-centered medical services, which has been gaining momentum since the introduction of the Patient Experience evaluation in 2017 [24]. Additionally, public health policy initiatives, such as the “in-depth consultations of over 15 minutes” program for patients with serious and non-curable diseases, have been in place since 2018 [25]. Despite these efforts, challenges still remain. According to a recent Organization for Economic Co-operation and Development (OECD) Health Statistics report, South Korea has one of the highest numbers of outpatient visits among OECD countries, with an average of 16 visits per year per patient [26]. However, patient experiences regarding communication with physicians remain relatively low. For example, 87.1% of patients reported that they could easily understand their doctor’s explanations, ranking South Korea 13th out of 19 countries, while only 81.8% felt they had the opportunity to ask questions or express concerns, ranking 13th out of 18 countries, the lowest among OECD nations [27]. Although approximately 80% of patients reported overall satisfaction, these findings highlight significant opportunities for improvement in achieving patient-centered care.

While Western healthcare systems have advanced patient-centered care, cultural factors in Asian countries, including South Korea, continue to influence patient-physician communication dynamics. In many Asian countries, including South Korea, the patient-physician relationship has traditionally been described as more hierarchical and paternalistic compared to Western settings [28, 29]. However, recent evidence suggests a growing orientation toward patient-centered care among providers in Asian clinical settings, reflecting broader shifts in healthcare expectations and policies [9, 23, 30, 31]. In addition, communication styles can also be a critical point. Physicians’ communication style can be understood as both a stable trait, reflecting consistent practice patterns, and a dynamic state influenced by patient demographics and interaction contexts [32, 33]. Patient communication styles also shape clinical interactions. Younger patients and those with higher socioeconomic status tend to engage more actively, while older patients often adopt a passive role [34–36]. Demographic, language and cultural concordance between patients and physicians can also influence communication effectiveness [37–39].

To enhance patient-centered care, both consultation time and communication styles must be optimized through a comprehensive approach. To date, studies on patient-physician communication in South Korea have primarily focused on patients’ perceptions and satisfaction rather than employing observational methods, leaving the actual communication processes during consultations largely unexplored. This gap highlights the need for a deeper understanding of communication dynamics in medical consultations. Moreover, this gap reflects a broader issue in East Asia, where patient centered care philosophies, widely embraced in Western healthcare systems, may not yet be fully integrated into local medical practices [10]. How critical challenges for clinicians, researchers, and policymakers in the region determine how to effectively implement patient-centered care through meaningful communication that meets patients’ needs and preferences in practice.

To address these gaps, this study analyzes real-world communication during outpatient consultations at a secondary-level general hospital in South Korea. Focusing on internal medicine and surgery departments, we use direct observation through audio recordings and apply the Roter Interaction Analysis System (RIAS), a widely validated framework for coding patient-physician communication. RIAS categorizes utterances into instrumental (task-oriented) and affective (socioemotional) domains, capturing both biomedical content and interpersonal expressions such as empathy, reassurance, and rapport-building [14]. Originally developed in Western settings, RIAS has been successfully adapted in various Asian contexts, including South Korea, demonstrating its cross-cultural applicability [40–43]. Unlike self-reported assessments of communication quality, our observational approach allows for empirical identification of communication patterns in time-limited clinical encounters. By examining how structural features, particularly consultation length and visit type, relate to physician–patient interaction dynamics, the study contributes to ongoing discussions about optimizing patient-centered care in high-volume health systems.

Previous cluster-analytic studies of clinical consultations, such as those by Roter et al. (1997) and Bensing et al. (2003) [14, 44], have identified recurring communication patterns between physicians and patients. These include the biomedical pattern, characterized by closed-ended questions and biomedical information exchange with limited affective content; the biopsychosocial pattern, which incorporates both biomedical and psychosocial dialogue and is often associated with greater emotional engagement; and the consumerist pattern, where patient-initiated questions and physician responses dominate, reflecting a more transactional interaction style. These empirically observed patterns align with conceptual models of physician–patient relationships such as those described by Emanuel and Emanuel (1992), who categorized relationships into paternalistic, informative, interpretive, and deliberative models [45]. Building on this foundational work, our study explores whether similar communication patterns emerge in South Korea’s outpatient context, where consultation time is often limited.

The aim of this study is threefold: (1) to identify patient physician communication patterns observed in South Korean general hospital consultations, (2) examine how consultation length relates to different communication patterns, and (3) investigate key factors influencing doctor patient communication. The specific research questions (RQ) are:

1. What communication patterns are observed in South Korean outpatient consultations?
2. How does consultation length differ across communication patterns?
3. What consultation characteristics and physician-level factors are associated with these communication patterns?

## Methods

### Study participants and data collection

This study is a secondary analysis of recorded medical consultations conducted at a public hospital in Seoul, South Korea, between 2016 and 2018. The hospital, as a secondary care facility, plays a significant role in providing specialized healthcare and managing chronic diseases for the urban population of Seoul. The consultations were audio-recorded in outpatient departments, covering general outpatient visits, chronic disease management, and specialized care. These recordings aimed to improve understanding of healthcare service, evaluate patient-centered care practices, and provide insights into clinical interactions in real-world settings. A total of 35 physicians and 520 patients participated in this study. After excluding cases in which the recording was interrupted or a proxy (rather than the patient) participated in the consultation, 510 recordings, or consultations, were included in the analysis.

### Study measures

Physicians’ age, medical experience, and seniority were self-reported via a brief demographic form developed for this study. This form did not include any psychometric or clinical items. Departments were categorized as internal medicine or surgery, following the classification system of the South Korean Hospital Association. Clinical environments were assessed for the presence of caregivers during visits, which included identifying third parties other than clinicians, such as nurses or caregivers. Consultations were classified as first-visit or follow-up visits. The length of consultation was measured as the actual minutes of the audiotape of the verbal interaction between the physician and patient, excluding any examination time.

### Ethical approval

This study was approved by the Institutional Review Board of Seoul National University Boramae Medical Center (IRB No. 20181112/10-2018-106/121) and Seoul National University (IRB No. E1911/001-004). It was conducted in accordance with the ethical principles of the Declaration of Helsinki. Patients were voluntary and were informed that their decision to participate would not affect their treatment. Written informed consent was obtained prior to each consultation. To protect confidentiality, all recordings were de-identified before analysis, and any identifying information was removed.

### Communication coding and analysis using RIAS

The recorded consultations were manually analyzed using the Roter Interaction Analysis System (RIAS), a widely recognized and validated method for coding clinical communication. Manual coding was conducted directly from audio recordings without transcription, using RIAS-specific coding software. The unit of analysis was the utterance, defined as the smallest distinguishable segment of speech to which a classification can be assigned [40]. Each utterance was coded into one of 41 mutually exclusive categories [46]. Across all consultations, we coded approximately 20,000 utterances using the RIAS protocol. RIAS functioned both as a coding scheme and a conceptual framework, based on a functional model of medical dialogue that separates instrumental (task-oriented) and affective (socioemotional) communication. No additional theoretical frameworks or sensitizing concepts were applied beyond the standard RIAS structure. RIAS-coded utterances were further grouped into nine subcategories according to established RIAS-based classification systems [14]. The RIAS framework includes 38 mutually exclusive codes for physicians and 31 for patients [47, 48]. For analytic clarity, these were aggregated into ten conceptually meaningful categories, as shown in Supplement Table 1 [14, 44].

**Table 1:** Descriptive characteristics of patients, physicians, and clinical environment

Characteristics	Category	*n*	%
**Patients**			
Gender	Men	315	61.8
	Women	195	38.2
**Physicians**			
Gender	Men	27	77.1
	Women	8	22.9
Age Group	30s	15	42.9
(Mean = 40.83, SD = 5.01)	40s	18	51.4
	50s	2	5.7
Medical Career (Years)	5–10	4	11.4
(Mean = 15.57, SD = 5.25)	10–15	12	34.3
	15–20	11	31.4
	> 20	8	22.9
Years of Service at Hospital	< 3 years	13	37.1
(Mean = 5.62, SD = 4.99)	3–5 years	5	14.3
	5–10 years	9	25.7
	10–15 years	7	20
	> 15 years	1	2.9
**Clinical Environment**			
Presence of Caregiver	Yes	126	24.3
	No	384	75.1
Consultation Type	First-visit	105	20.5
	Follow-up visit	405	79.5
Medical Department	Internal Medicine	394	77.3
	Surgery	116	22.7
**Consultation Time (in minutes)**		**Mean**	**SD**
Total		5.11	2.8
First-visit		7.01	3.59
Follow-up visit		4.61	2.31

Beyond standard RIAS coding, we analyzed physician interruptions during patient speech, a common feature of clinical interactions [49]. Interruptions were categorized into seven types: (1) turn interruptions, (2) facilitative interruptions, (3) confirmations of partner information, (4) expressions of disagreement, (5) clarification-seeking, (6) humorous or joking interruptions, and (7) monitoring interruptions to confirm information [49] (Supplement Table 2). Although interruptions often carry negative connotations in English, prior studies suggest they may support rapport-building or conversational flow in clinical communication. We also captured back-channel responses, such as “hmm” or “okay”, which signal attentiveness and encourage the speaker to continue [24].

**Table 2:** Mean number and proportion of utterances per RIAS category, stratified by speaker (physician vs. patient)

	Physician			Patient		
RIAS Category	Mean (SD)	Range	% Utterance	Mean (SD)	Range	% Utterance
***Total Utterance (n)***	35.98 (18.7)	8.0–132.0	100	26.82 (16.5)	3.0–130.0	100
***1. Instrumental Domain***	24.49 (12.6)	5.0–95.7	68.1	15.26 (10.2)	0.0–92.5	56.9
(1) Biomedical exchange						
a. Asking questions	6.08 (11.1)	0.0–62.5	16.9	2.9 (6.6)	0.0–57.1	10.9
b. Information giving	13.04 (15.7)	0.0–83.1	36.2	10.5 (14.4)	0.0–90.0	39
(2) Psychosocial exchange						
a. Asking questions	0.49 (3.6)	0.0–27.3	1.4	0.1 (1.1)	0.0–20.0	0.46
b. Information giving	0.3 (2.8)	0.0–27.6	0.8	1.3 (5.2)	0.0–66.7	4.73
(3) Partnership building	0.51 (2.2)	0.0–10.5	1.4	0.2 (1.3)	0.0–15.0	0.73
(4) Directions	3.64 (7.0)	0.0–31.6	10.1	0.3 (2.1)	0.0–20.0	1.1
***2. Affective Domain***	11.48 (12.6)	4.4–100.0	31.9	11.6 (10.4)	7.5–100.0	43.1
(1) Rapport building	6.94 (9.3)	0.0–60.0	19.3	5.7 (7.2)	0.0–78.6	21.1
(2) Showing Concern and Optimism	1.11 (4.4)	0.0–25.0	3.1	2.2 (5.1)	0.0–28.6	8.2
(3) Social Behavior	3.17 (6.7)	0.0–41.7	8.8	3.6 (6.9)	0.0–50.0	13.4
(4) Disagreements	0.26 (2.7)	0.0–26.3	0.7	0.1 (1.3)	0.0–15.4	0.4

Percentages reflect the relative frequency within each speaker’s total utterances

Note: Utterances are the primary unit of analysis in RIAS, defined as a single complete thought. Percentages reflect the proportion of each utterance category relative to the speaker’s total utterance (i.e., physician or patient). Totals for domains (Instrumental/Affective) include all relevant subcategories

In addition, to capture conversational dynamics, we calculated a physician-dominance score, defined as the ratio of physician to patient utterances. Higher dominance scores indicate greater physician control over the conversation, characterized by more physician speech relative to patient speech. In contrast, lower dominance scores reflect a more balanced or patient-centered exchange [50].

To assess inter-coder reliability, approximately 20% of consultations (*n* = 95) were independently coded by a second trained coder, and Cohen’s Kappa coefficient was calculated to measure agreement. To account for differences in consultation length, we calculated both the average number of utterances per consultation and the proportion of each utterance category relative to the speaker’s total utterances.

### Statistical analysis

Descriptive analyses were conducted to summarize physicians’ characteristics and clinical environments. K-means cluster analysis was employed to classify patient-physician communication patterns based on utterance, enabling the exploration of their relationship with consultation time. Three communication patterns were adopted from prior studies, focusing on utterances categorized as biomedical and psychosocial questions, as well as information exchange [14, 44]. This approach aligns with the widely recognized continuum of patient-centered and physician-centered communication [51].

The optimal number of communication patterns was determined using the average silhouette method [52], which assesses how well each data point fits within its assigned communication pattern compared to others. Following clustering, ANOVA and Tukey’s post-hoc tests were conducted to examine differences in utterances, dominance, control, and consultation time across the identified patient-physician communication patterns. All statistical analyses were performed using R software (version 4.4.1), with the “FactoMineR” [53] and “factoextra” [54].

To identify the factors associated with communication patterns, a multinomial logistic regression was conducted, with the biomedical pattern set as the reference category. Predictor variables included physician and patient gender, physician career length, years of hospital service, presence of caregivers, visit type (first-time vs. follow-up), medical department, and consultation time. Odds ratios (ORs) and 95% confidence intervals (CIs) were calculated for the likelihood of adopting biopsychosocial or consumerist communication patterns relative to the biomedical pattern.

## Results

### Patient, physician, and consultation characteristics

Table 1 presents the descriptive statistics of the study participants and consultation characteristics. The patient sample consisted of 61.8% men and 38.2% women, while the physician sample was predominantly men (77.1%). The average age of physicians was 40.83 years (SD = 5.01), with most falling within the 30s (42.9%) and 40s (51.4%) age groups. Physicians had an average of 15.57 years of medical experience (SD = 5.25), with 34.3% having 10 to 15 years of experience and 31.4% having 15 to 20 years. Regarding the clinical environment, caregivers were present in 24.3% of consultations, while 75.1% of consultations involved only the patient and physician. Most consultations were follow-up visits (79.5%), with first-time visits comprising 20.5%. Internal medicine accounted for most consultations (77.3%), while surgery consultations represented 22.7%.

Consultation time varied significantly by visit type. The overall mean consultation time was 5.11 min (SD = 2.8). First-visit consultations were significantly longer, averaging 7.01 min (SD = 3.59), whereas follow-up consultations averaged 4.61 min (SD = 2.31). These findings highlight the structured nature of follow-up consultations, which are less time-consuming compared to initial visits (Table 1).

The analyzed results revealed descriptive findings of patient-physician communication across 510 consultations using the RIAS methodology. The average number of patient-physician utterances and their proportions are summarized in Table 2. In each consultation, physicians spoke between 8 and 132 times, with a mean of 35.98 utterances, while patients spoke between 3 and 130 times, averaging 26.82 utterances. Overall, the number of patient utterances was significantly lower than that of physicians, indicating a statistically significant difference.

Overall, biomedical exchange’ was the most common utterance of the physician, especially information giving utterances accounting for 36.2% of total utterances, followed by rapport building (19.3%) and biomedical questions (16.9%). Overall biomedical exchange, biomedical question asking and information giving accounted for 53.1% of all physicians’ utterances. When physician communication patterns were categorized into instrumental (task-based) and affective (psychosocial) domains, instrumental communication comprised 68.1% of physician utterances, significantly exceeding affective communication (31.9%). For patients, biomedical information giving was the most frequent utterance (39.0%), followed by rapport building (21.1%) and social behavior (13.4%). Patient communication patterns were also predominantly instrumental (56.9%), although their affective communication percentage (43.1%) was notably higher than that of physicians (31.9%) (Table 2).

On average, physicians interrupted patients 3.4 times per consultation (SD = 3.2). While nearly 49% of consultations included fewer than two interruptions, a small subset (7.1%) involved more than 10 interruptions. Physicians’ interruptions of patient utterances were primarily observed when patients expressed concerns or sought reassurance. These interruptions resembled facilitative interruptions, where physicians anticipated patient responses, but often reflected their own concerns or anxieties.

### Communication patterns

**Fig. 1:**
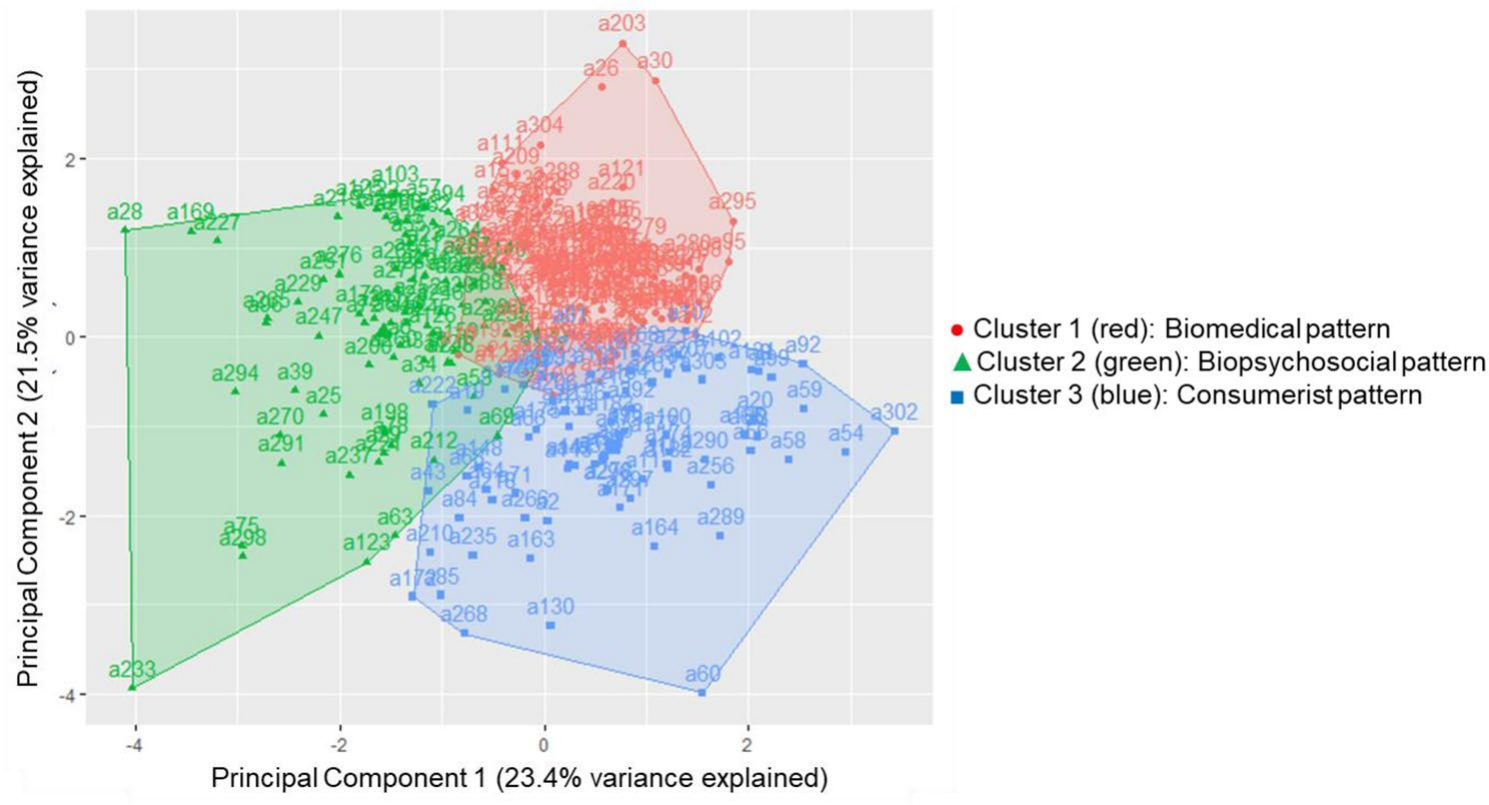
Cluster visualization of patient-physician communication patterns. Note. Principal component analysis was used to reduce dimensionality of RIAS-coded utterance variables. K-means clustering revealed three distinct communication patterns: Biomedical (Cluster 1, red circles), Biopsychosocial (Cluster 2, green triangles), and Consumerist (Cluster 3, blue squares). The axes represent the first two principal components, explaining 44.9% of the total variance. Each point represents one outpatient consultation

Using the silhouette method, three distinct patient–physician communication patterns were identified through K-means clustering (Fig. 1). Following the classification approach by Bensing, Roter, and Hulsman (2003) [14], the resulting patterns were descriptively labelled as “biomedical,” “biopsychosocial,” and “consumerist.” The distinguishing characteristics of each were based on the mean frequency of key communication variables within each communication pattern.

The biomedical pattern was characterized by the highest total utterance count from both physicians and patients, as well as the most frequent physician interruptions (mean = 6.9 per consultation). Instrumental communication dominated this pattern, with physicians asking a high number of biomedical questions (28.22 utterances per consultation) and patients contributing substantial biomedical information (55.69 utterances per consultation). In contrast, affective behaviors such as rapport-building and social talk utterances were minimal. The biopsychosocial pattern exhibited the fewest interruptions (mean = 1.72) and the lowest overall utterance count, but was marked by greater emphasis on affective communication, including rapport-building and social behavior, features consistent with a patient-centered approach. The consumerist pattern shared the biomedical pattern’s emphasis on information exchange but featured more patient-initiated questions and routine conversation within the affective domain, though affective behaviors were less frequent than in the biopsychosocial pattern (Tables 3 and 4).

**Table 3:** Utterances by identified patient-physician communication patterns

	Overall	Biopsychosocial pattern		Consumerist pattern		Biomedical pattern		F	*P*
Physician									
**Total Utterance(n)**	35.98	28.24	a	37.91	a	48.2	a	39	***
**Interruption(n)**	3.49	1.72	a	3.2	a	6.9	a	110.3	***
***Instrumental Domain (%)***	68.07	63.6	a	73.8	d	71.06	d	22.1	***
(1) Biomedical exchange									
Asking questions	16.89	14.3	a	9.32	a	28.22	a	113.6	***
Information giving	36.23	32.98	b	49.8	d	30.12	b	48.7	***
(2) Psychosocial exchange									
Asking questions	1.36	2.02	b	0.5	d	0.92		5.2	**
Information giving	0.83	0.89		1.19		0.38		1.7	-
(3) Partnership building	1.43	1.55		1.23		1.38		0.6	-
(4) Directions	10.12	10.6		10.27		9.12		1.3	-
***Affective Domain (%)***	31.91	36.4	a	26.17	d	28.92	d	22.2	***
(1) Rapport building	19.28	21.5	b	14.48	a	19.54	b	15.6	***
(2) Showing Concern and Optimism	3.1	3.47		3.51		2.06		3.2	*
(3) Social Behavior	8.82	10.71	a	7.83	d	6.29	d	14	***
(4) Disagreements	0.71	0.72		0.35		1.03		1.3	-
**Patient**									
**Total Utterance (n)**	26.82	20.35	a	26.23	a	38.95	a	44	***
***Instrumental Domain (%)***	56.91	49.63	a	58.19	a	68.87	a	51.5	***
(1) Biomedical exchange									
Asking questions	8.84	4.6	b	20.37	a	6.31	b	193	***
Information giving	39.04	33.94	c	30.56	c	55.69	a	86	***
(2) Psychosocial exchange									
Asking questions	0.46	0.45		0.71		0.25		1.2	-
Information giving	4.73	6.02		3.4		3.57		3.7	*
(3) Partnership building	0.73	0.86		0.52		0.67		0.7	-
(4) Directions	1.1	1.47	b	0.39	d	1.07		4.3	**
***Affective Domain (%)***	43.09	50.37	a	41.81	a	31.13	a	51.5	***
(1) Rapport building	27.42	32.27	a	26.14	a	19.84	a	27.6	***
(2) Showing Concern and Optimism	2.6	2.3		2.9		2.88		0.6	-
(3) Social Behavior	12.66	15.48	a	12.34	a	7.9	a	20	***
(4) Disagreements	0.4	0.32		0.43		0.51		0.4	-

* Comparison of patterns by using Tukey’s test (*p* < 0.05)

a: Significantly differ from two other patterns

b: Significantly differ from Consumerist pattern

c: Significantly differ from Biomedical pattern

d: Significantly differ from Biopsychosocial pattern

**Table 4:** Multinomial logistic regression: factors associated with communication patterns (Reference = Biomedical Pattern)

		B	Exp(B)	95% CI	
Biopsychosocial pattern	**Characteristics of patients and physicians**				
	Patient gender (Ref: male)	0.08	1.09	0.59	1.99
	Physician gender (Ref: male)	-0.15	0.86	0.4	1.84
	Medical career as physicians	0.02	1.02	0.94	1.1
	Length of service in the hospital (years)	-0.03	0.97	0.89	1.05
	**Characteristics of clinical environment**				
	Presence of caregiver (Ref: None)	-0.29	0.75	0.36	1.55
	First-time visit (Ref: Follow-up visit)	-0.71*	0.49	0.24	0.56
	Medical department (Ref: Internal medicine)	0.1	1.1	0.51	2.38
	Consultation time	-0.01***	0.89	0.80	0.95
Consumerist pattern	**Characteristics of patients and physicians**				
	Patient gender (Ref: male)	0.08	1.09	0.69	2.66
	Physician gender (Ref: male)	-0.15	0.86	0.31	1.65
	Medical career as physicians (years)	0.08*	1.08	1.01	1.18
	Length of service in the hospital (years)	-0.07	0.93	0.85	1.02
	**Characteristics of clinical environment**				
	Presence of caregiver (Ref: None)	-0.55	0.58	0.27	1.25
	First-time visit (Ref: Follow-up visit)	-0.69	0.5	0.22	1.12
	Medical department (Ref: Internal medicine)	-0.55	0.58	0.26	1.28
	Consultation time	-0.003	0.99	0.90	1.01

### Comparison of consultation time across communication patterns

The biopsychosocial pattern was the most prevalent, accounting for 48.9% of consultations, followed by the consumerist (27.1%) and biomedical (23.9%) patterns. Consultation duration differed significantly across these patterns (F = 30.50, *p* < 0.001): biomedical consultations were longest (mean = 6.78 min), followed by consumerist (5.36 min) and biopsychosocial (4.07 min) consultations (Table 3). Pattern distribution also varied by visit type. Among first-visit consultations, the consumerist pattern was most common (47.7%), followed by biopsychosocial (30.8%) and biomedical (21.5%) patterns. In contrast, follow-up consultations most frequently exhibited the biopsychosocial pattern (53.7%), with biomedical (24.6%) and consumerist (21.7%) patterns less common. Notably, none of the 35 physicians adhered exclusively to a single pattern; each demonstrated at least two distinct styles across visits, suggesting dynamic variation in utterance.

### Related factors of patient-physician communication patterns

Multinomial logistic regression was used to examine factors influencing communication patterns (Table 4). In the biopsychosocial pattern, follow-up visits compared to first-time visits (OR = 0.49, 95% CI: 0.24–0.56) and shorter consultation times (OR = 0.89, 95% CI: 0.80–0.95) were related to biopsychosocial pattern. For the consumerist pattern, physicians with longer medical careers were more likely to adopt this communication style (OR = 1.08, 95% CI: 1.01–1.18). Other variables, such as physician and patient gender, the presence of caregivers, and medical department, showed no significant impact. The explanatory power of the model (Nagelkerke R²) was 0.24.

## Discussion

This study identified three distinct patterns of patient-physician communication in South Korean clinical settings: biomedical, consumerist, and biopsychosocial. The biomedical pattern, characterized by instrumental communication and minimal affective interaction, reflected a high level of physician control, with an average of 6.9 interruptions per consultation and the longest consultation time (6.78 min). The consumerist pattern, also emphasizing instrumental communication, featured active patient inquiries and high patient control, with physicians acting as technical consultants and fewer interruptions (3.2 on average). The biopsychosocial pattern balanced instrumental and affective communication, fostering mutuality and relationship-building, with affective communication accounting for 36.4% of physicians’ and 50.37% of patients’ utterances. Consultations in this pattern were the shortest, averaging 4.07 min.

This study provides valuable insights into the practical application of patient-centered care in South Korean clinical settings and offers broader implications for other healthcare systems where patient-centered communication has yet to be fully integrated into medical practice. First, the study results highlight the relationship between consultation time and the communication patterns between physicians and patients. The biomedical pattern was associated with longer consultation times, allowing for more extensive biomedical exchange while maintaining strong physician control. Conversely, the biopsychosocial pattern demonstrated that patient-centered communication can occur within shorter consultations, suggesting that efficient use of consultation time may enhance the quality of communication rather than being solely dependent on its duration. In addition, considering Zerubavel’s (1979) concept of consultation time being determined by task completion rather than a fixed schedule supports this observation [55]. Physicians’ control often dictated the conclusion of consultations, emphasizing instrumental (task-oriented) interactions.

However, the implications of consultation length must be understood within South Korea’s specific healthcare landscape. As Bensing, Roter, and Hulsman (2003) noted, consultation time is shaped not only by healthcare system characteristics but also by cultural expectations regarding medical care [14]. Many South Korean patients prefer large general hospitals with advanced medical technology, leading to high patient loads and time constraints [56]. These pressures, exacerbated by structural inefficiencies in the national healthcare system, result in efficiency-driven workflows that prioritize rapid diagnosis and treatment over in-depth patient engagement. Under these conditions, physicians struggle to engage in affective communication, particularly within the biomedical pattern, which is instrumental (task-oriented) [57, 58]. Even when physicians attempt to balance institutional demands with affective engagement, the limited duration of consultations may still result in suboptimal patient experiences. Additionally, patients may experience communication challenges and feel pressured when expressing their concerns or asking questions to the physician [59].

Second, a notable finding of this study is that the biopsychosocial pattern accounted for a higher proportion of consultations compared to findings from other countries. Specifically, the biopsychosocial pattern accounted for 48.9% of consultations. While similar studies found biopsychosocial communication in 27% of U.S. consultations [44] and 13% in the U.S. and the Netherlands [14], South Korean consultations showed a significantly higher proportion (48.9%). However, these interactions occurred within a much shorter timeframe than in Western contexts, where biopsychosocial consultations lasted an average of 16.1 min [60], compared to 4.07 min in South Korea. This suggests that while South Korean physicians engage in patient-centered communication, systemic constraints on consultation length limit the depth of these interactions.

Third, these findings provide empirical support for theoretical frameworks on patient-physician relationships, particularly the role of dominance and control in shaping communication patterns [61, 62]. Control behaviors emerged as key parameters distinguishing the identified patterns. This study provides empirical confirmation of theoretical patient-physician relationship models in South Korean clinical settings. Emanuel & Emanuel (1992) proposed classifications of patient-physician relationships based on power dynamics, suggesting that relationships are shaped by (1) who sets the consultation agenda, (2) the degree to which patient values are incorporated, and (3) the functional role of the physician [45]. In line with previous studies using this model, our research extends its application to an underexplored East Asian context, particularly South Korea.

Given the hierarchical nature of South Korean medical culture [63], it is unsurprising that the biomedical pattern accounted for nearly one-third of all consultations. This pattern featured the highest level of physician speech dominance and instrumental communication, with biomedical exchange making up the majority of both physician and patient speech. Emotional communication, such as small talk and rapport-building, was minimal. Physician-led questioning dominated interactions, while patient-initiated inquiries were infrequent. The strong paternalistic nature of this communication pattern was further evidenced by high physician control, including frequent interruptions during consultations.

Notably, this study also underscores the interactive nature of patient-physician communication and reveals how communication patterns are shaped. High levels of patient inquiries, rapport-building, and expressions of concern, alongside physicians’ extensive provision of biomedical information in biopsychosocial patterns, demonstrate the interconnected nature of these interactions. The consumerist pattern exemplified how patient demand for information prompted physicians to allocate more time to information provision. While physicians’ utterances reflect individual traits and situational factors, patients’ active participation also plays a crucial role in shaping interaction. Educational initiatives for physicians to enhance communication skills are essential, but patients must also be empowered to engage effectively during consultations. This dual approach can foster more meaningful exchanges and support the development of mutual understanding in clinical settings.

While we identified the biopsychosocial pattern as more aligned with patient-centered care due to its balance of instrumental and affective communication, we recognize that patient-centeredness is ultimately defined by the degree to which care aligns with each patient’s values, preferences, and needs. Some patients may experience biomedical-focused interactions as entirely appropriate and satisfactory. However, our categorization draws on established conceptual frameworks that emphasize the importance of addressing both biomedical and psychosocial domains to fully support patient-centered care [3, 64]. Emotional communication and rapport building, even when limited, can create space for trust and shared decision-making, which are central to a patient-centered orientation [65]. Evidence from South Korea is still limited; however, a validated Korean Patient-Centered Care tool identifies key domains such as information sharing, physical comfort, emotional support, and responsiveness to patient values [66].

The findings of this study have several implications for clinical practice. First, improving patient-centered communication does not necessarily require longer consultations but rather an optimized approach to time management and communication strategies. Training programs for physicians should focus on techniques that enhance biopsychosocial interactions even within time-constrained environments. Second, to optimize patient-centered communication, healthcare policies should consider adjusting reimbursement rates based on the need for more time-consuming consultations. For example, consultations that involve complex medical conditions, detailed patient education, or psychosocial issues requiring significant affective communication could be reimbursed at higher rates to ensure sufficient time for these important discussions. By aligning financial incentives with the need for more thorough communication, such a policy change could encourage healthcare providers to engage more fully with patients, enhancing the quality of the patient-physician relationship and overall care experience. Finally, empowering patients to actively participate in consultations, such as by encouraging them to ask questions and express concerns, may further enhance patient-physician communication and overall satisfaction. Addressing these factors may help bridge the gap between systemic constraints and the delivery of high-quality, patient-centered care. These findings suggest that consultation time alone is not sufficient to promote patient-centered communication. Rather than uniform consultation lengths, policies should support flexible time allocation based on patient complexity and communication needs. Embedding such flexibility within institutional structures could enhance patient-provider interactions, particularly in high-volume health systems like South Korea.

### Study limitations

While the study offers valuable insights, several limitations should be acknowledged. First, the data were collected from a single medical facility, limiting the ability to analyze the influence of organizational factors on communication patterns. Second, due to patient confidentiality protection, demographic information beyond gender (e.g., age and socioeconomic status) was unavailable, preventing an in-depth analysis of how these factors relate to communication patterns. Third, while this study focused on objective communication patterns, future research should explore patients’ subjective experiences and perceptions to better understand how communication patterns influence patient satisfaction and outcomes. Despite these limitations, this study offers valuable contributions by empirically analyzing real patient-physician communication in South Korean clinical settings, rather than relying on patient self-reports. Fourth, the study sample may not fully represent the general South Korean patient population. As data were collected from a single secondary care hospital in an urban area, the findings may not be generalizable to other healthcare settings such as primary care clinics, tertiary hospitals, or rural health centers. Differences in healthcare delivery systems and patient characteristics across regions and institutions could influence communication patterns and consultation dynamics. Fifth, our classification of communication patterns in terms of patient-centeredness was based on the content and relational features observed through RIAS coding, rather than direct input from patients regarding their preferences, experiences, or satisfaction. While our approach was grounded in widely accepted theoretical models of patient-centered care, it may not fully capture individual variations in what patients consider appropriate or desirable communication. Future research should integrate patient-reported outcomes to assess how different interaction styles align with perceived needs and values across diverse populations.

## Conclusion

This study identified three distinct communication patterns, with the biopsychosocial pattern being the most prevalent. Consultation length varied significantly across these patterns, with biopsychosocial consultations being the shortest, challenging the assumption that longer visits inherently facilitate patient-centered communication. Additionally, our findings suggest that physician career length, follow-up visits, and systemic factors play a role in shaping communication patterns, highlighting the importance of institutional and cultural contexts in medical interactions. These findings contribute to the broader discourse on patient-centered care by demonstrating how communication styles reflect both physician tendencies and systemic constraints. Given the increasing emphasis on patient experience, healthcare policymakers and practitioners should consider strategies to optimize communication quality within the constraints of consultation length.

## Data Availability

Data are available from the corresponding author upon reasonable request.
